# Pralatrexate induced durable response in a relapsed/refractory peripheral T-cell lymphoma patient with a history of autologous stem cell transplantation: Case report of a patient followed-up over 3 years under pralatrexate treatment: Erratum

**DOI:** 10.1097/MD.0000000000020260

**Published:** 2020-05-01

In the article, “Pralatrexate induced durable response in a relapsed/refractory peripheral T-cell lymphoma patient with a history of autologous stem cell transplantation: Case report of a patient followed-up over 3 years under pralatrexate treatment”,^[1]^ which appears in Volume 98, Issue 30 of *Medicine*, the statement “The authors have no conflicts of interests to disclose” should appear as “ should appear as “The authors received assistance with the article processing charge from myTomorrows (Impatients N.V. company).”

## References

[1] Merdin A, İskender D, Ulu BU Pralatrexate induced durable response in a relapsed/refractory peripheral T-cell lymphoma patient with a history of autologous stem cell transplantation: Case report of a patient followed-up over 3 years under pralatrexate treatment. *Medicine*. 2019 98;e16482. 31348254 10.1097/MD.0000000000016482 PMC6709046

